# Analysis of a Functional IL-6 Gene Polymorphism in HLAB27 Associated and Intermediate Uveitis Gives New Insight in Disease Pathogenesis and Commonality with Other Autoimmune Diseases

**DOI:** 10.1155/2015/174062

**Published:** 2015-11-10

**Authors:** Lindner Ewald, Langner-Wegscheider Beate, Sarny Stephanie, Renner Wilfried, El-Shabrawi Yosuf

**Affiliations:** ^1^Department of Ophthalmology, Medical University Graz, Auenbruggerplatz 4, 8036 Graz, Austria; ^2^Department of Ophthalmology, General Hospital Klagenfurt, St. Veiter Strasse 47, 9020 Klagenfurt, Austria; ^3^Clinical Institute of Medical and Chemical Laboratory Diagnostics, Medical University Graz, Auenbruggerplatz 15, 8036 Graz, Austria

## Abstract

*Purpose*. Interleukin 6 (IL-6) plays a crucial role in both adaptive and innate immunity. The rs1800795 gene polymorphism of IL-6 is associated with various autoimmune diseases, like multiple sclerosis. *Methods*. 134 patients with HLAB27 positive iridocyclitis, 84 patients with intermediate uveitis, 132 controls, and 65 HLAB27 positive controls were recruited for the present case-control study. Main outcome measures were genotype distribution and allelic frequencies determined by polymerase chain reaction. *Results*. The frequency of carriers of the minor allele for rs1800795 was significantly higher in patients with intermediate uveitis compared to controls (*p* = 0.04; OR: 1.46; CI: 1.02–2.11). Frequencies of the minor allele for rs1800795 did not differ significantly in patients with HLAB27 associated uveitis when compared to controls (*p* > 0.05). *Conclusion*. These findings further deepen our understanding of the commonality between multiple sclerosis and intermediate uveitis. Given the functionality of the investigated polymorphism, new pathophysiological insights are gained that help to evaluate possible therapeutic targets.

## 1. Introduction

The treatment of endogenous uveitis can be very challenging because of side effects or lack of efficacy of standard immunomodulatory treatment. Biologicals, such as selective cytokine inhibitors, may offer a more tailored therapeutic venue [1]. However the lack of knowledge on the pathogenesis of uveitis and the differences in between the various uveitis entities, for instance, regarding the specific cytokine patterns, has so far made that approach difficult. Candidate gene analyses give new insights in the pathogenesis of uveitis and may help to find possible therapeutic targets. We for instance have recently found a genetic variant of interleukin 2 receptor alpha (IL2RA) associated with intermediate uveitis (IU), but not with HLAB27 acute anterior uveitis (HLAB27 AAU) [2], suggesting IL2RA as a possible therapeutic target in IU. Treatment with biologicals has to be evaluated with care and separately in each and every disease. TNF alpha-blockers used for the therapy of rheumatoid arthritis have been shown to worsen [3] and to initiate multiple sclerosis (MS) [4, 5]. Thus, the application of biologicals should be based on a thorough knowledge of the pathogenesis which is supported by gene analyses.

Interleukin 6 (IL-6) plays a pivotal role in the immune processes of many diseases. IL-6, together with transforming growth factor- (TGF-) *β*, induces differentiation of IL-17-producing T helper cells (Th17) [6] that play a crucial role in the development of various autoimmune diseases including systemic lupus erythematosus [7], Behçet's disease [8], or rheumatoid arthritis [9]. On the other hand IL-6 exerts also anti-inflammatory effects via the induction of interleukin-1 and tumor necrosis factor alpha antagonists [10] and it has been shown that IL-6 deficiency leads to increased expression of other cytokines [11]. Tocilizumab blocks IL-6 signaling [12] and has been approved by the Food and Drug Administration (FDA) for use in selected patients with rheumatoid arthritis [13]. Results in spondyloarthritis were disappointing [14], which highlights the role to assess targets in each disease separately.

The IL-6 gene polymorphism (-174 G/C, rs1800795), which is functional [15, 16] with the G allele increasing IL-6 production, has been found to be associated amongst others with toxoplasmic retinochoroiditis [17] and multiple sclerosis [18]. Interestingly, this polymorphism is not associated with ankylosing spondylitis [19]. Given the fact that IU is the most common form of uveitis in multiple sclerosis patients [20] and since HLAB27 AAU is the most common extra-articular manifestation in ankylosing spondylitis [21], we wanted to evaluate the role of rs1800795 in IU and HLAB27 AAU.

## 2. Materials and Methods

Study participants were recruited at the Department of Ophthalmology, Medical University Graz, Austria. All participants were Caucasian and were living in the same geographic region in the south of Austria. After detailed explanation of the nature and possible consequences of the study, the patients signed a written informed consent. The study was conducted according to the tenets of the Declaration of Helsinki (2013) and was approved by the ethics committee of the Medical University of Graz.

Gender, age at presentation, age at onset of uveitis, systemic disease association, number of flares, duration of flares, duration between flares, and prevalence of severe ocular complications were recorded in order to characterize the study population. Significant cataract (greater than or equal to 2+ opacity) or secondary glaucoma was documented as complications. SUN criteria [22] were used to define HLAB27 AAU and IU. HLAB27 AAU patients were examined by a rheumatologist for clinical and radiographic signs and symptoms of spondyloarthropathy. In case of symptoms of inflammatory back pain or other symptoms compatible with spondyloarthropathy radiographs of the sacroiliac joints and the spine were made. In order to rule out radiologic signs in accordance with a possible diagnosis of MS such as presence and distribution of white matter lesions, MRI of the brain was obtained in all patients suffering from IU. In case of neurological symptoms patients were examined by a neurologist and a lumbar puncture with testing for oligoclonal bands was performed.

As controls 143 random, unrelated, healthy individuals attending our department for reasons other than ocular inflammation were included. Exclusion criteria were any history of intraocular inflammation, arthritis, lower back pain, autoimmune diseases, or malignancy. None of the controls showed any signs of past uveitis episodes (e.g., residual pigment on lens) in slit-lamp examination. The past medical history was collected following a routine questionnaire. Of course it cannot be ruled out that the controls will eventually develop autoimmune diseases or malignancies in the future. All control subjects were genotyped for HLAB27. Eleven HLAB27 positive controls, together with 54 HLAB27 positive healthy unrelated blood donors, whose DNA was provided by the Department of Blood Serology and Transfusion Medicine, served as the HLAB27 positive control group.

## 3. Genetics

DNA was extracted from peripheral lymphocytes using the nucleic isolation kit: QIAamp DNA Mini and Blood Kit (QIAGEN; Netherlands) following the manufacturers protocol and stored at −20°C.

Genotype determination was performed using high-resolution melting curve analysis on the LightCycler 480 PCR system. The samples were amplified in duplicate 20 *μ*L reactions using the Light Cycler 480 High Resolution Melting Master kit (Roche Diagnostics, Vienna, Austria) and analyzed on a LC480 instrument I (Roche Diagnostics GmbH, Mannheim, Germany). The final reaction mixture contained 1x Master Mix, 3 mM MgCl_2_, 4 *μ*M forward and reverse primer, and 50 ng of genomic DNA. For PCR the following cycling conditions were chosen: one cycle of 95°C for 10 minutes followed by 45 cycles of 95°C for 10 seconds, 60°C for 15 seconds, and 72°C for 20 seconds. The amplicons were then denaturated at 95°C for 1 minute, cooled down to 40°C for 1 minute, and then melted from 65°C to 95°C with 25 signal acquisitions per degree. To detect sequence variations the Gene Scanning Software version 1.5 (Roche Diagnostics GmbH, Mannheim, Germany) was used. Samples were automatically grouped because of their melting curves using the Auto Group mode.

## 4. Statistics

Statistical analysis was performed using PASW 22.0 (SPSS Inc., Chicago, IL). Means were compared using Mann-Whitney *U* test. Proportions of groups were compared by the *χ* ^2^ test. Odds ratio (OR) and 95% confidence interval (95% CI) were calculated by logistic regression. The criterion for statistical significance was *p* ≤ 0.05. Hardy-Weinberg equilibrium has been calculated using HW Diagnostics-Version 1 beta (Fox Chase Cancer Center, Philadelphia, PA).

## 5. Results

For this study 134 patients with HLAB27 AAU (59 female (44.03%)), 84 patients with IU (49 female (58.34%)), 132 HLAB27 negative controls (38 female (28.79%)), and 65 HLAB27 positive controls (31 female (47.70%)) were enrolled. The mean age was 44.81 ± 14.70 for patients with HLAB27 AAU, 30.82 ± 16.66 for patients with IU, 35.56 ± 12.10 for HLAB27 negative controls, and 37.75 ± 4.02 for HLAB27 positive controls. Differences in age between the groups were tolerated since polymorphisms do not change with age.

For rs1800795 an association has been found with the C allele significantly more prevalent in IU patients (*p* = 0.04, allelic OR: 1.46 (1.02–2.11)). No significant differences were found in genotype or allele distribution between patients with HLAB27 AAU and HLAB27 positive or negative control subjects (*p* ≥ 0.05; Table 1). Controls were divided into HLAB27 positive and negatives in order to rule out any confounding effect of HLAB27.

Observed genotype frequencies of both polymorphic markers were in accordance with the Hardy-Weinberg equilibrium (data not shown). No association was found between the investigated polymorphism and the investigated ocular parameters depicted in Table 2. Two patients with IU had MS and they were homozygous for the C allele.

## 6. Discussion

This is the first study to demonstrate an association of the rs1800795 gene variant and IU. Given the known effect of this polymorphism on IL-6 levels and its association with related diseases our findings give new insights in the pathophysiology of IU and are especially interesting concerning a potential use of IL-6 as therapeutic target.

The C allele at rs1800795 was associated with lower IL-6 levels. Lower plasma levels of IL-6 in healthy individuals [16] and lower LPS-stimulated IL-6 production* ex vivo* were found. Furthermore the C allele was found to bind nuclear protein less avidly than the G allele, which demonstrates possible molecular mechanisms of the investigated alterations [15].

Effects of rs1800795 have been found in a wide variety of conditions including cancer [23], psychiatric diseases [24], arthrosclerosis [25], and even sport performance [26]. In several autoimmune diseases an association with this polymorphism has been reported. Interestingly some diseases are associated with the G allele and some with the C allele. Besides its proinflammatory properties IL-6 has been shown to act anti-inflammatory as well by the induction of interleukin-1 and tumor necrosis factor alpha antagonists [10]. In our study we found the C allele to be the risk allele for IU which is in line with findings in type-1-diabetes [27], Hashimoto's thyroiditis [28], and most importantly MS [18]. Autoimmune conditions are apparently very distinct in their development which leads to two considerations. First, a specific therapy which addresses this heterogeneity might be very competitive compared with the standard regimen in terms of efficacy and safety. Secondly, a targeted treatment which is beneficial for one condition might be harmful in another as this was seen with TNF alpha-blockers [3–5]. Regarding anti-IL-6 treatment the FDA lists nervous system problems including multiple sclerosis as possible serious side effects of tocilizumab [29]. Given the commonality between MS and IU and taking into account the findings presented here and in previous studies, which suggest parallel pathways of MS and IU, we suggest that evaluation of anti-IL6 in IU should be carried out with caution. A phase 2 clinical trial with Sarilumab, a high affinity IL-6 receptor antibody, in noninfectious uveitis will be completed this year (NCT01900431) and its results are awaited with high interest.

The following potential limitations should be kept in mind, when interpreting our results. Only one gene polymorphism in the IL-6 gene was analyzed, so we cannot rule out that the true causative variant lies somewhere else within the LD block; therefore further sequencing of the IL-6 gene may reveal further associations of other variants. Secondly, as our study population was of European descent our findings might not apply to populations other than Caucasian. Thirdly, we did not measure IL-6 levels in our samples. Furthermore, only two patients had intermediate uveitis and multiple sclerosis. A larger number of patients suffering from both diseases could help to discover further genetic commonalities.

## 7. Conclusion

In conclusion we found that the functional IL-6 polymorphism rs1800795 is associated with IU but not with HLAB27 AAU. Our findings further highlight the commonality between IU and MS and give new insights in the pathogenesis of IU. Since rs1800795 is known to be functional our results may help to evaluate a possible therapy targeting IL-6.

## Figures and Tables

**Table 1:** Distribution of the investigated gene polymorphisms in patients and controls.

	HLAB27 AAU	IU	HLAB27 negative controls	HLAB27 positive controls	Controls combined
	(*n* = 134)	(*n* = 84)	(*n* = 132)	(*n* = 65)	(*n* = 197)
rs1800795					
G/G	40 (29.9%)	19 (22.6%)	51 (38.6%)	24 (36.9%)	75 (38.1%)
G/C	76 (56.7%)	50 (59.5%)	62 (47.0%)	31 (47.7%)	93 (47.2%)
C/C	18 (13.4%)	15 (17.9%)	19 (14.4%)	10 (15.4%)	29 (14.7%)

AAU: acute anterior uveitis.

IU: intermediate uveitis.

**Table 2:** Baseline ocular and systemic parameters.

Patient characteristics	HLAB27 AAU	IU
	(*n* = 134)	(*n* = 84)
Mean age of onset ± SD (years)	35.64 ± 14.10	25.91 ± 14.59
Mean number of flares ± SD	6.93 ± 7.80	4.24 ± 7.17
Mean duration of flares ± SD (weeks)	4.00 ± 2.74	6.02 ± 6.39
Mean duration between flares ± SD (months)	21.19 ± 18.38	11.29 ± 12.10

Secondary cataract	21 (15.67)	11 (13.10)
Secondary glaucoma	5 (3.73)	2 (2.40)

Ankylosing spondylitis	57 (42.54)	0 (0.00)
Juvenile idiopathic arthritis	1 (0.75)	0 (0.00)
Undifferentiated spondyloarthritis	18 (13.43)	0 (0.00)
Reactive arthritis	5 (3.73)	0 (0.00)
Crohn's disease	1 (0.75)	1 (1.20)
Psoriatic arthritis	12 (9.00)	0 (0.00)
Multiple sclerosis	0 (0.00)	2 (2.40)

HLAB27 AAU: HLAB27 associated acute anterior uveitis.

IU: intermediate uveitis.

SD: standard deviation.

Values are *n* (%) unless otherwise indicated.
